# Dural Arteriovenous Fistulas in the Cavernous Sinus: Clinical Research and Treatment

**DOI:** 10.5402/2011/453834

**Published:** 2011-08-02

**Authors:** Akira Kurata, Sachio Suzuki, Kazuhisa Iwamoto, Kuniaki Nakahara, Makoto Sasaki, Chihiro Kijima, Madoka Inukai, Katsutoshi Abe, Jun Niki, Kimitoshi Satou, Kiyotaka Fujii, Shinichi Kan

**Affiliations:** ^1^Department of Neurosurgery, Kitasato University School of Medicine, 1-15-1 Kitasato, Sagamihara, Kanagawa 228-8555, Japan; ^2^Department of Radiology, Kitasato University School of Medicine, Kanagawa 228-8555, Japan

## Abstract

*Introduction*. The purpose of this paper is to clarify the clinical course, with the dural carotid cavernous fistula (CCF), featuring a pallet of symptoms, paying special attention to radiological findings. *Methods*. Seventy-six consecutive patients with dural CCFs were investigated in detail, all of whom were defined by angiography. *Results*. The most common initial symptom was diplopia in 47 patients (62%) and the most frequently observed on arrival were type II, featuring cranial nerve palsies followed by the classical triad in 27, and then type I only with cranial nerve palsies. The time until admission with type I (mean: 6.7 W ± 6.0) was significantly shorter than that with type II (mean: 25.1 W ± 23.5). Branches from bilateral carotid arteries widely inflowing into bilateral carotid cavernous sinus were present in 30 (39%), 20 (26%) of which also demonstrated direct inflow into the intercavernous sinus. type I and II had more multiple venous drainage routes as compared with type III (classical triad only on arrival) and IV (initial development of the classical triad followed by cranial nerve palsy). *Conclusion*. In our series of dural CCF patients, the most common initial symptom was cranial nerve palsy, mostly featuring multiple venous drainage including cortical drainage. Such palsies should be added to the classical triad as indicative symptoms. Bilateral carotid arteries often inflow into cavernous and intercavernous sinuses, which should be taken into account in choice of therapeutic strategy.

## 1. Introduction

Dural arteriovenous fistulas are not rare in Japan, especially examples involving the cavernous sinus. However, reports of large series are few [1], and the clinical entity is not widely known. The classical triad [2, 3] of pulsating exophthalmos, conjunctival chemosis, and pulsatile-innitus are well-established clinical symptoms of the disease but are not usually present in the majority of the patients as early indicators. The D-CCF may therefore be overlooked, especially the bilateral type. The purpose of this paper is to clarify the clinical course with a pallet of symptoms in patients with D-CCF, with especial attention to results of radiological studies.

## 2. Methods

142 patients with dural arteriovenous fistulas were consecutively experienced in our institute from October 1985 to June 2010, all of whom were defined by angiography. Seventy-six (54%) demonstrating involvement of the cavernous sinus (CS) are the subjects of this paper. All but two were diagnosed after 1990. Sixty-one (80%) were female and 15 (20%) were male, with ages ranging from 40 to 77 years, with an average of 64 years. Radiological findings with bilateral carotid angiography and clinical courses of symptoms were evaluated in detail for each case.

Data were analysed by the Student *t*-test (two-tailed) for paired values, using the statistical program Stat Mate III for Windows version 3.19. Differences were considered statistically significant at *P* < 0.05.

## 3. Treatments

Eleven patients were conservatively treated, three aged more than 70 years old. Five of the others were classified as Barrow type B [4], and the remaining three demonstrated slow and mild inflow into the cavernous sinus. All these conservative cases showed no aggressive symptoms like decrease of visual acuity, severe retro-orbital pain, or cranial nerve palsies. Radiological findings revealed no cortical venous reflux. Endovascular surgery was performed for the other 65 patients, transarterial embolization (TAE) for inflowing meningeal branches from the external carotid arteries being the initial approach in all cases. Until 1996, polyvinyl alcohol particles ranging from 150 to 200 microns in diameter were mainly used as embolic material. Since 1997, platinum particles of 100–200 microns and platinum coils were employed.

To avoid complications, provocative testing using 2% xylocaine raging from 30 mg to 60 mg was repeatedly performed before embolization [5]. If cranial nerve palsy developed, the catheter tip was advanced further. If the catheter could not be advanced, a platinum coil was used as embolic material. Eighteen patients required transvenous embolization (TVE) following TAE because of residual cortical venous drainage or aggressive symptoms. One of 2 who received stereotactic radiosurgery (SRS) in addition to TAE and TVE has already been reported [6]. The other received SRS after TAE without TVE because of no available transvenous access routes.

## 4. Results

### 4.1. Clinical Course and Radiological Examination

#### 4.1.1. Initial Symptoms (Table 1)

The most common initial symptom was diplopia, evident in 47 patients (62%) (Table 1). In three cases it was transient, and in the others it persisted until admission. Unilateral third cranial nerve palsy was the most frequent, found in 25, followed by unilateral sixth cranial nerve palsy in 14. One of the classical triad, at least was the next most frequent symptom, encountered in 27. Retro-orbital or/and forehead pain was recognized in 24, this being unilateral in 22. In all except 5, this was transient in the range of 2 hours to 1 month (average 12 days). Two demonstrated persistence for 1 month and the other three for a comparatively long periods with repeated remission and aggravation for 5 M, 5 M, and 7 M until admission. In the majority of patients with cranial nerve palsies, the initial diagnoses were aneurysm, unknown origin and diabetes mellitus. In those with conjunctival chemosis, the initial diagnosis was conjunctivitis. In patients with bilateral conjunctival chemosis and proptosis, the diagnosis was allergic conjunctivitis. Retro-orbital or/and forehead pain was initially diagnosed as trigeminal neuralgia and migraine. With cranial nerve palsies combined with retro-orbital pain, the initial diagnosis was the Tolosa-Hunt syndrome.

#### 4.1.2. Relationships between Inflowing Arteries, Affected Sinus, and Clinical Symptoms (Table 2)

Bilateral carotid angiography was conducted for all 76 patients (Table 2). In fifty seven patients (75%), branches from the bilateral carotid arteries were involved. Bilateral type D by Barrow's classification [4] was most frequent in 38 of 57. Bilateral CS was noted in 30 (39%), with the intercavernous sinus (ICS) affected in 20 (26%). Ipsilateral CS and ICS were impacted in 20 (26%). Ipsilateral CS only was evident in 26 (34%) overall and in 12 of 57 of the bilateral type. ICS involvement was apparent in 40 of 76 patients. 

 Unilateral clinical symptoms were recognized in 64 of the 76. Bilateral carotid arteries participated in 46 and contralateral carotid arteries only in one. The total 12 with bilateral clinical symptoms comprised 4 with bilateral cranial nerve palsies, 1 with bilateral classical triad, 3 with bilateral cranial nerve palsies and the bilateral classical triad, and three with the unilateral classical triad and contralateral cranial nerve palsy. One patient showed ipsilateral cranial nerve palsies with orbital and contralateral orbital pain. All except one featured bilateral carotid artery participation. Nine were classified as bilateral type D by Barrow, two as type DB, and the other one as type D. In seven, bilateral CS and ICS were affected, one with bilateral CS, two with ipsilateral CS and ICS. Only two featured ipsilateral CS. Multiple venous drainage routes were common radiological findings in all of 4 cases presenting with bilateral cranial nerve palsies. The superior ophthalmic vein (SOV) was the drainage route in three of these, but pulsating exophthalmos and conjunctival chemosis were not shown.

#### 4.1.3. Relationships between Clinical Symptoms and Venous Drainage (Table 3)

The clinical symptoms and the time course before arrival at hospital were divided into four groups, as shown in Table 3: type I: cranial nerve palsies only on admission; type II: cranial nerve palsies preceded by classical symptoms; type III: classical symptoms only on admission; type IV: classical symptoms preceded by cranial nerve palsies. The most common symptoms on arrival were type II, in 27 of 76 patients, all except 6 of whom presented more than 3 months after the initial signs. The next was type I in 25 cases, all but two arriving less than 3 months after development of the initial symptom. The time until admission in type I ranged from 4 days to 6 months (mean: 6.7 W ± 6.0), which was significantly short (*P* < 0.01, *G* test:) as compared with type II (mean: 25.1 W ± 23.5). The symptoms on arrival were limited to classical triad (type III) in only 13 patients. Eleven patients initially developed the classical triad followed by cranial nerve palsies (type IV). 

 In type I, drainage routes ranged from one to eight (average: 2.6 ± 1.3). Cortical venous drainage was present in 9 of the 25 (33%). In 22 (88%), the inferior petrosal sinus (IPS) participated as a venous drainage route. SOV was the venous drainage route in 11 (44%) and the pterigoid plexus (PP) in 8 out of the 9.

 In type II, drainage routes ranged from one to six (mean: 2.3 ± 1.4). Cortical venous drainage was present in 12 of 27 (44%). SOV was venous drainage in 24 (90%) and IPS in 11. PP did not attribute as a venous drainage route.

 In the 13 type III cases, drainage routes ranged from 1 to three (mean: 1.7 ± 0.9). All except four had only one venous drainage route, the SOV. Cortical venous drainage was only present in one (7.7%). 

 In the 11 of type IV, drainage routes ranged from one to six (mean: 2.2 ± 2.0). Cortical venous drainage was present in 2 (18.2%). SOV and/or inferior ophthalmic vein (IOV) participated as venous drainage routes in all and IPS in 4.

The numbers with venous drainage and cortical vein involvement among types I~IV did not show statistically significant variation.

### 4.2. Complications Caused by Endovascular Surgery and Patients Outcome

No technical complications occurred with TAE. Cranial nerve palsies were avoided by 2% xylocaine testing preceded by embolization. Unfortunately, one patient died of lung infarction caused by a deep venous thrombosis from the lower limb. In only one patient, the sixth cranial nerve palsy persisted despite complete disappearance of the dural arteriovenous fistula after TAE, as defined by long-term follow-up magnetic resonance angiography (MRA). One other patient with an isolated sinus having only the petrosal vein via the superior petrosal sinus venous drainage route, who rejected additional open surgery, developed subarachnoid hemorrhage followed by SRS. This patient only showed disturbance of consciousness as a severe disability (SD) on the Glasgow-Outcome Scale 4/9.

 In 4 of eighteen patients treated with additional TVE, the sixth cranial nerve palsy developed between two and four days after the TVE treatment. In three patients this resolved completely 2 months, 3 months, and 11 months later. 

In 9 of 11 patients conservatively followed-up, all except one of fistulas were completely occluded on MRA after 1 month to 13 years 5 months (average: 5 years 3 months). One patient still exhibited residual fistulas on follow-up angiography 1 year after the initial symptoms. Another was complicated with central retinal thrombosis during the follow-up period and the other 2 patients were lost to follow up. 

 In 47 patients treated with only TAE, all but two were followed by MRA. In 43 (90%) of 45, complete obliteration was established on MRA after periods ranging from 1 month to 13 years 8 months (average: 4 years 6 months). The remaining two patients had residual fistulas after 6 year 2 months and 2 year. Both of the patients refused additional treatment because of the lack of symptoms. 

 In all 18 patients treated with TAE followed by TVE, fistulas were completely occluded on follow-up MRA after 1 month to 3 years 6 months (average: 2 years 3 months) after the treatment. In one patient receiving TAE, TVE, and SRS, follow-up MRA 10 years and 8 months after the treatment showed complete disappearance of the fistula.

## 5. Discussion

The clinical symptoms pulsating exophthalmos, bruit, and conjunctival chemosis are well known to be a classical triad indicative of carotid cavernous fistula (CCF) [2, 3], related to venous drainage routes. Walker and Allegre [3] in fact reported that lack of exophthalmos is very rare, while Taniguchi et al. [7] focused on absence of a bruit as the principal cause of misdiagnosis. When the classical triad is missing, patients often have been initially misdiagnosed. However, we have already presented evidence that dural CCF patients usually do not show the classical triad, sometimes presenting only with cranial nerve palsies, with many venous drainage routes including the cortical [8]. In the present dural CCF series, cranial nerve palsies were the most common initial symptoms, the majority being persistent in contrast to an earlier report [9]. 

On the other hand, at least one of the classical triad was the initial symptom in only 36% of cases. Newton and Hoyt [9] reported unilateral head pain to be the commonest early symptom usually passed off as an unusual migraine attack. In this series also, unilateral headache was frequently recognized mostly transient with a duration of less than 1 month). The time course of clinical symptoms was of interest. Cranial nerve palsy followed by classical symptoms (type II) was most frequent followed by cranial nerve palsy alone.

Multiple venous drainages route may be attributable for symptoms limited to cranial nerve palsies because dispersion of the arterial flow will conceal the classical triad. In type II, instead of PP, the SOV contributed the major venous drainage route presumably related to occlusion of the PP over time. The mechanism is obscure but may involve thrombi. With type III and type IV, the SOV and/or the IOV were the main venous drainage routes without any cortical contribution perhaps because of secondary occlusion of the latter.

 Serious complications such as intracerebral hemorrhage are very rare with dural AVFs of the cavernous sinus, the majority of patients exhibiting an extremely benign clinical course [10, 11]. Exceptionally, some may feature decrease of visual acuity including central retinal thrombosis and cortical venous reflux evident on angiography. Spontaneous regression in dural CCF is not uncommon and was noted in 5 of 11 cases reported by Newton and Hoyt [9], 19 of 26 by Sasaki et al. [10], and three of 18 by Vinuela et al. [12]. Recently, TVE has been proposed as a more appropriate curative treatment than transarterial embolization (TAE), but it may result in serious outcomes like embolic stroke as reported by Halbach et al. [13], especially if performed without prior arterial flow reduction by TAE. Yamashita et al. [14] reported complication occurring in 7 of 16 patients undergoing TVE one featuring epidural extravasation from perforation of the inferior petrosal sinus and the other 6 transient aggravation of symptoms (chemosis and sixth/third cranial nerve palsy in three each). Major complications are particularly associated with cortical venous reflux. Araki et al. [15] reported extravasation from the uncal vein during TVE for SOV and IOV via the IPS and emphasized the importance of obliterating cortical venous drainage as early as possible, even when the reflux is small. Watanabe et al. [16] described a dural CCF patient in whom the cavernous sinus received normal cortical drainage from the insular vein. Post-embolization MRI showed cerebral infarction caused by congestion in the area of the posterior insular vein because the anticipated drainage of the insular vein via the uncal vein had not occurred. Nakamura et al. [17] emphasized preservation of Sylvian venous flow on finding the affected cavernous sinus to receive not only the shunted flow but also the Sylvian venous drainage in three cases (12%) of 26 dural CCFs treated. In all of our series treated with TAE, complications fortunately did not occur. However, in 4 (22%) of eighteen undergoing additional TVE, sixth cranial nerve palsy developed between two and four days after the treatment. While it fortunately resolved completely after less than 3 months in two cases and 11 months in one, it improved and persisted in the other. Aihara et al. [18] reported deterioration of oculo-motor dysfunction in two of 9 patients with dural AVF involving the cavernous sinus after TVE due to two different causes. High intrasinus pressure caused by blockage of the drainage pathway resulted in the cranial nerve palsy in one. Implanted coils directly compressed the cranial nerve in the other. In our series, sixth cranial nerve palsy developed after several days due to thrombosis around the platinum coils inserted into the posteromedial part of the cavernous sinus. Minimal insertion of coils appears essential to avoid such complications. Targeting TVE with a minimum of coils may be optimal treatment, but only after TAE to reduce the arterial inflow and the size of the affected lesions 

In our series, bilateral type D by Barrow's classification accounted for half (50%). Ipsilateral CS was affected in only 34 %. Takahashi et al. [19] reported ICS involvement in 5 of 8 consecutive patients, all of which could be successfully treated by the whole cavernous sinus packing via the TVE. However, they mentioned difficulty with target embolization via the IPS in cases with fistulas at the bilateral cavernous sinuses or posterior intercavernous sinus. In dural CCF patients, bilateral angiography would appear to be essential given our findings for bilateral cavernous sinuses and also ICS. In dural CCF patients with wide involvement of the sinuses (type of bilateral CS and bilateral CS and ICS), especially with a cortical venous reflux, TAE first for reduction of inflow initially has been recommended as a reasonable treatment strategy to avoid serious complication because of the comparatively nonaggressive nature of the disease [20]. Kupersmith et al. [21] described complications occurring in 5 of 38 dural CCF cases undergoing TAE, but IBCA treatment was an additional factor in four of these. Particles may be safer than liquid emboli and in our series of TAE using only particles, no complications occurred. Repeated provocative testing and care of dangerous anastomosis are also important. Comparative long follow-up MRA (average: 4 years 6 months) showed complete obliteration in 93% but additional TVE is needed for residual fistulas, especially in cases still having cortical venous drainage. This can be curative as evidenced by complete occlusion of fistulas in all 18 of our patients treated with TAE followed by TVE. One further received stereotactic radiosurgery (SRS) as reported earlier [6].

## 6. Conclusion

In our series of dural CCF patients, the most common initial symptom was cranial nerve palsy, most featuring multiple, including cortical, venous drainage. Such palsy should be added to the classical triad as indicative symptoms. Bilateral carotid arteries often inflow into cavernous and intercavernous sinuses, which should be taken into account in choice of therapeutic strategy.

## Figures and Tables

**Table 1:** Summary of initial symptoms and diagnosis in 76 patients with dural CCF.

Initial symptoms	Number of cases	Initial diagnosis
Orbital or forehead pain	24	Trigeminal neuralgia Migraine Tolosa-Hunt syndrome
Diplopia (cranial nerve palsies)	47*	Aneurysm Unknown cause Diabetes mellitus
III	25	
III, VI	5	
VI	14	
IV, VI	1	
III, IV, VI	1	
Classical symptoms	27	Conjunctivitis Sclerosis
Triad	2	
PE	1	
PE + CC	6	
PE + PT	1	
CC	11	
PT	6	

*Excluded 1 case with transient diplopia.

III: third cranial nerve palsy.

IV: fourth cranial nerve palsy.

VI: sixth cranial nerve palsy.

triad: PE, CC and PT.

**Table 2:** Summary of afferents in 76 patients with dural carotid cavernous fistulas.

	Affected sinus	
Barrow type*	Ipsilateral cavernous sinus	Bilateral cavernous sinus
	(Direct inflow into ICS)	
A	—	—
B	3(0)	0(0)
C	3(0)	0(0)
D	13(4)	0(0)
D&D	16(11)	22(17)
D&B	7(3)	4(1)
D&C	2(1)	2(2)
B&B	1(0)	1(0)
C&C	1(1)	1(0)
Total	46(20)	30(20)

Barrow type*.

Type A fistulas are direct shunts between the internal carotid artery and the cavernous sinus.

Type B, C, and D fistulas are dural shunts.

B between meningeal branches of the internal carotid artery and the cavernous sinus.

C between meningeal branches of the external carotid artery and the cavernous sinus.

D between meningeal branches of both internal and external carotid arteries and the cavernous sinus, ICS: inter-cavernous sinus.

**Table 3:** Time course of clinical symptoms and venous drainage in 76 patients with dural CCF.

Types of clinical course	No. cases	Initial symptoms forehead or retro-orbital pain (at the same time)	Time until admission (mean ± SD)	Ave. No. VD (% of CVD)
(i) Cranial nerve palsies Only	25	11(3)	4 D–6 M (6.7 W ± 6.0*)	2.6 ± 1.3 (33%)
(ii) Cranial nerve palsiesfollowed by classical symptoms	27	6(0)	2 M–24 M (25.1 W ± 23.5)	2.3 ± 1.4 (44%)
(iii) Classical symptoms Only	13	4(0)	1 W-14 M (19.1 W ± 18.7)	1.7 ± 0.9 (7.7%)
(iv) Classical symptoms followed by cranial nerve palsies	11	4(2)	1 M–7 M (11.9 W ± 9.0)	2.2 ± 2.0 (18.2%)

M: month; W: week; D: day; Ave: average; VD: venous drainage; CVD: cortical venous drainages.

*P <  0.01 (compared with Type II): Student *t*-test.
